# Impact of Glycemic Trajectory and Variability on Ischemic Stroke Prognosis and Exploration of Its Mechanisms: A Comprehensive Review

**DOI:** 10.3390/healthcare14152327

**Published:** 2026-08-01

**Authors:** Xiaofei Wu, Meng Jin, Ge Geng, Yujia Jin, Jinhua Chen

**Affiliations:** 1 Department of Nursing, The Second Affiliated Hospital of Zhejiang University School of Medicine, Hangzhou 310009, China; 2503049@zju.edu.cn; 2 Department of Neurology, The Second Affiliated Hospital of Zhejiang University School of Medicine, Hangzhou 310009, China; 22318453@zju.edu.cn (M.J.); 12318500@zju.edu.cn (Y.J.); 3 The Fifth Clinical Medical College, Henan University of Chinese Medicine, Zhengzhou 450052, China; 2022154257@hactcm.edu.cn

**Keywords:** ischemic stroke, glycemic trajectory, glycemic variability, continuous glucose monitoring, prognosis

## Abstract

The association between glycemic trajectories and variability with new-onset stroke and stroke outcomes remains poorly understood. Previous studies have predominantly focused on the absolute levels of single-point glucose measurements and emphasized mean glucose levels, overlooking individual differences in baseline levels and variability. This study describes prior research exploring the relationship between glucose trajectories and variability with post-stroke hemorrhagic transformation, short- and long-term clinical outcomes, post-stroke pain, post-stroke depression, and post-stroke cognitive impairment. It also discusses approaches to achieving precise and standardized measurement of glucose trajectories and variability, as well as existing methods for effectively improving glucose fluctuations. The findings suggest that abnormal glucose trajectories and high variability are independently associated with adverse stroke outcomes. However, significant gaps remain regarding standardization of variability metrics, integration of continuous glucose monitoring into acute stroke care, and the lack of prospective interventional data. Future research should prioritize the establishment of unified assessment standards and well-designed prospective studies to translate these findings into clinical practice.

## 1. Introduction

Globally, stroke ranks as the second leading cause of death among noncommunicable diseases (NCDs) and the third leading cause of combined mortality and disability (measured in disability-adjusted life years—DALYs) [1]. From 1990 to 2021, the global burden of stroke increased substantially in low- and lower-middle-income countries, with 69.0% attributable to metabolic risk factors. This disproportionate societal burden significantly shortens productive life expectancy [2]. Stroke manifests as ischemic and hemorrhagic phenotypes, with ischemic stroke constituting the predominant type [3], accounting for 87% of all stroke subtypes in the United States and 63% globally [4]. Vascular causes of ischemic stroke include large-artery disease, cardioembolic stroke, and small-vessel disease, with the latter also being the most common cause of intracerebral hemorrhage [5]. Ischemia induces direct neuronal injury, excessive reactive oxygen species (ROS) production, and inflammatory activation, leading to rapid brain tissue pathology, blood–brain barrier disruption, and progressive microvascular damage [6,7,8]. This triggers a cascade of adverse outcomes including cerebral edema, hemorrhagic transformation, neuroinflammation, infarct enlargement, and neurological deficits. Therefore, reducing and preventing stroke-induced neurological damage is crucial for lowering mortality and disability risks in the global population.

The INTERSTROKE study indicates that abnormal blood glucose accounts for 3.9% of population-attributable risk in stroke [9]. Risk factor profiles differ between ischemic stroke subtypes—macroangiopathic atherosclerotic and cerebrovascular disease—but abnormal blood glucose remains a major shared risk factor for both [10]. Extensive research data support the association between abnormal blood glucose and microcirculatory dysfunction, which triggers key molecular events in atherosclerosis through inducing oxidative stress and inhibiting nitric oxide (NO) bioactivity [11,12]. Furthermore, emerging evidence indicates that ischemic stroke in diabetic patients exhibits significantly heightened Toll-like receptor 4 (TLR4)-mediated neuroinflammation compared to non-diabetic ischemic stroke [13,14,15]. Thus, optimal glycemic control represents a critical step in enhancing functional recovery in stroke patients.

Approximately 16–24% of diabetic patients face stroke risk [16]. Insulin resistance and hyperinsulinemia predispose to abnormal fatty deposition in intracranial and extracranial arteries, leading to macrovascular complications [17]. The STOP NIDDM study demonstrated that regular use of acarbose significantly reduces stroke incidence [18]. A multicenter randomized controlled trial from the UK (UKPDS study) highlighted the importance of intensive glycemic control, showing that a 1% reduction in hemoglobin A1c (HbA1c) lowers stroke risk by 4% [19]. Studies including the Insulin Resistance Intervention in Stroke (IRIS), Glucose and Insulin in Stroke Trial Pilot (GIST), and Insulin in Acute Ischemic Stroke (INSULINFARCT) all demonstrated the coexistence of abnormal glucose levels and stroke, as well as the therapeutic efficacy of glucose intervention in diabetic stroke patients [20,21,22].

Previous studies have predominantly focused on the impact of static blood glucose values at specific time points on IS prognosis. Regarding variability, inconsistencies exist in fluctuation assessment criteria and differences in repeat measurement intervals. Moreover, most previous studies examining the relationship between blood glucose and IS prognosis treated glucose as a discrete endpoint, using single-time-point measurements as the exposure factor. They rarely considered the degree of change, overlooking glucose variability or reversibility. However, the process of glucose change—particularly its deterioration or improvement—may exert distinct effects on IS prognosis. Current research has paid scant attention to this aspect, failing to comprehensively evaluate the potentially complex relationship between glucose oscillations and IS outcomes.

This paper will elaborate on glucose oscillations as a representation of blood glucose fluctuations, current metrics for glucose oscillations (variability and trajectory levels), the impact of blood glucose trajectories and variability on short- and long-term IS prognosis, and targeted improvement measures. In contrast to static markers such as HbA1c or admission glucose, glycemic trajectory analysis captures longitudinal patterns of glucose change over time. While HbA1c reflects average glucose levels over the preceding 2–3 months and admission glucose represents a single time-point measurement, trajectory modeling can identify distinct subpopulations with different patterns of glucose evolution, thereby offering additional prognostic information beyond static or average values.

## 2. Methods

This is a narrative review aimed at synthesizing and critically evaluating the current literature on the mechanisms and management of glycemic trajectories and variability in relation to stroke occurrence and prognosis. To identify relevant publications, we conducted a systematic search in the electronic databases PubMed, Web of Science, and Google Scholar for articles published from inception to March 2026. The search strategy employed a combination of keywords and Medical Subject Headings (MeSH) terms related to ‘glycemic trajectories’, ‘glycemic variability’, ‘glycemic fluctuation’, ‘glycemic oscillation’, ‘stroke’, ‘cerebrovascular accident’, and ‘stroke prognosis’. The reference lists of retrieved articles were also manually screened for additional relevant studies. Both clinical studies (including observational studies and randomized controlled trials) and preclinical animal studies were considered for inclusion. Emphasis was placed on original research articles and high-quality reviews published in English. The selection process was iterative and involved screening titles and abstracts, followed by full-text review of potentially eligible articles. Data related to the epidemiology, pathophysiology, and therapeutics of glycemic trajectories and glycemic variability were extracted and thematically synthesized to provide a comprehensive overview of the field. Given the narrative and integrative nature of this review, no formal quality assessment or risk of bias analysis of individual studies was performed, and the synthesis aimed to present a balanced perspective on existing knowledge and emerging concepts. After screening titles and abstracts for relevance and excluding duplicate publications and non-English articles, approximately 130 references were included for full-text review and thematic synthesis. When conflicting findings were encountered, priority was given to studies with larger sample sizes and more rigorous designs.

## 3. Glucose Oscillation

Oscillatory glucose is a term used in the literature to describe the daily fluctuations in plasma glucose concentration in normal or diabetic individuals. Blood glucose dynamically fluctuates over time rather than being a static indicator. Compared to persistently elevated glucose levels, glucose fluctuations pose greater harm. Glucose fluctuations between 5–15 mmol/L every 6 h exhibited significantly increased risk of adverse cardiovascular events compared to those maintaining continuous glucose levels of 10 or 15 mmol/L, suggesting that the magnitude and frequency of glucose oscillations may be more deleterious than chronic stable hyperglycemia alone [23]. Recent cross-sectional evidence from 200 type 2 diabetes patients suggests high glucose variability correlates with elevated fracture risk and reduced bone turnover rate [24]. Glucose oscillations can induce increased inflammatory factors through reactive oxygen species and NF-κB activation, stimulating monocyte adhesion and upregulating transendothelial transport protein expression [25]. Compared to sustained hyperglycemia, frequent and large fluctuations in glucose levels are more likely to induce endothelial dysfunction and oxidative stress, accelerating the progression of atherosclerosis. Ceriello et al. demonstrated that glucose oscillations specifically trigger elevated plasma 3-nitrotyrosine and 24 h urinary free 8-iso prostaglandin F2α (PGF2α) excretion rates—both recognized markers of oxidative stress [23]. Furthermore, the Diabetes Control and Complications Trial (DCCT) demonstrated that intensive insulin therapy groups exhibited hypoglycemia rates ranging from 10% to 30%, with severe hypoglycemia frequency increasing exponentially during blood glucose reduction [26]. Hypoglycemic events are considered one cause of increased cardiovascular events in diabetes and are closely associated with heightened risks for a series of adverse clinical outcomes in stroke patients. Research indicates that reducing blood glucose fluctuations can decrease both the duration and frequency of hypoglycemic episodes [27,28]. Glucose oscillations exert harmful effects on diverse cell types, including pancreatic β-cells [29], cardiomyocytes, neurons [30], renal cells, and endothelial cells [31], contributing to macro- and microvascular complications.

Multiple non-pharmacological and pharmacological strategies have been proposed to reduce glucose oscillations in human plasma. Dietary modifications, exercise, and weight loss represent the primary therapeutic approaches for controlling fluctuations. Intermittent scanning continuous glucose monitoring reduces glycemic variability by promoting behavioral changes through self-awareness of glucose oscillations [32]. A study utilizing CGM found that an extremely low-carbohydrate, high-fat breakfast reduced postprandial glucose fluctuations [33]. For obese populations, Farabi et al. demonstrated that exercise significantly impacts circadian oxidative stress and nocturnal glucose variability in obese individuals with type 2 diabetes and impaired glucose tolerance [34]. Another randomized crossover trial concurrently demonstrated that both aerobic and eccentric exercise reduce blood glucose variability in healthy individuals via inflammatory cytokine mediation [35]. Beyond glucagon-like peptide-1 analogues and dipeptidyl peptidase-4 inhibitors, which significantly influence blood glucose variability in type 2 diabetes mellitus (T2DM) patients [36], the development of rapid-acting and long-acting insulins has also positively impacted glycemic oscillation control. To date, studies in diabetic dogs and patients have separately validated that degludec insulin reduces hypoglycemic episodes and postprandial blood glucose fluctuations, suggesting its potential efficacy in controlling variability [37,38]. A self-controlled study involving 49 obese patients demonstrated that sleeve gastrectomy improves early glycemic homeostasis and reduces glycemic variability by suppressing post-operative growth hormone secretion [39]. Sodium-glucose cotransporter 2 (SGLT2) inhibitors, beyond controlling blood glucose, significantly reduce glycemic variability in untreated type 2 diabetic patients [40]. Finally, advances in diabetes monitoring and treatment technologies have enabled the identification of glycemic oscillations as an emerging target for improving overall diabetes management, aiming to flatten postprandial and fasting glucose curves to levels comparable to non-diabetic subjects.

## 4. Parameters for Evaluating Glucose Oscillation

There is no standardized quantitative metric for glycemic variability. Short-term and long-term glucose fluctuations, measured by changes in blood glucose amplitude, are categorized based on measurement intervals. Short-term glycemic variability reflects intraday and interday glucose oscillations. Parameters used in the literature to measure variability include standard deviation (SD), average real variability (ARV), coefficient of variation (CV), mean independent of mean (VIM), mean amplitude of glucose excursions (MAGE), M-value, J-index, mean absolute difference (MAD), mean absolute glucose (MAG), and continuous overall net glycemic action (CONGA)n. Each metric possesses distinct characteristics and applicable scenarios. Previous studies have differed in the metrics used to measure glycemic variability, with the coefficient of variation (CV) and mean amplitude of glycemic excursions (MAGE) being the predominant indicators. Moreover, the time intervals between measurements were not fixed across studies, ranging from days to years. In addition, individual glycemic fluctuations are influenced by factors such as physical activity and diet, as well as by reduced physiological reserve, functional decline, disease-related depletion, and more frequent use of glucose-lowering medications. Furthermore, the study populations varied across different investigations. These factors may collectively contribute to the discrepant findings observed in previous studies.

SD reflects the dispersion of measurements around the mean and is sensitive to low sampling frequencies. It is a commonly used expression of glucose variability that is easy to calculate and is included in nearly all articles addressing comprehensive variability assessment. Hirsch suggested that in T1DM, SD should not exceed the mean/2, while in T2DM, this parameter should be limited to mean glucose/3 [41]. CV, a standardized variable, enables direct comparisons between study groups. The ARV index averages absolute differences between consecutive measurements and may serve as a reliable indicator of time-series variability. However, SD, CV, and ARV partially depend on the mean and its temporal changes, which may remain unaddressed even after mean adjustment. VIM is a variability measure independent of the mean level and is sample-specific. A prospective cohort study from two tertiary stroke centers in Athens and the United States indicated that MAG negatively correlated with the likelihood of neurological improvement during hospitalization in stroke patients (OR, 0.14; 95% CI, 0.02–0.75; *p* = 0.022), but no association was found with 3-month clinical outcomes [42]. A 2020 retrospective cohort study using Korean National Health Insurance data, involving 624,237 diabetic patients aged ≥ 20 years, demonstrated a dose–response relationship between blood glucose VIM and stroke risk [43]. In a 2020 study of 170 patients diagnosed with severe acute stroke and admitted to a neurocritical care unit, mean follow-up of 2 years revealed that compared to mean blood glucose levels, blood glucose SD (OR, 1.14; 95% CI, 1.02–1.28, *p* = 0.028),MAGE (OR, 1.38; 95% CI, 1.04–1.83, *p* = 0.027) were significantly associated with the risk of death within 3 months in patients with severe acute stroke, indicating that a larger range of blood glucose drift suggests poorer disease prognosis [44]. A 2024 prospective study involving 17,789 participants from the Kailuan study examined the association between variability in selected metabolic syndrome parameters and the risk of total stroke and its subtypes in hypertensive patients. Results showed that a higher number of parameters with elevated glycemic variability coefficients was associated with increased risks of total stroke and ischemic stroke subtypes [45].

The J-index incorporates standard deviation in measuring glycemic variability, partially correcting the imperfect relationship between mean glucose and standard deviation. MAD, MAG, and (CONGA)n are parameters derived from continuous glucose monitoring analysis. MAD is based on five self-monitored blood glucose readings over 24 h [46]. MAG is the sum of differences in the continuous glucose curve per 24 h divided by the number of hours between the first and last glucose measurements [47]. The (CONGA)n assessment requires a complete tracking period and is defined as the standard deviation of differences. For each glucose data point observed after the first *n* hours, the difference between the current glucose and the glucose *n* hours prior is determined, with an analysis window of 24 h − *n*. The choice of time lag *n* depends on the clinical question being addressed. For example, in a 24 h monitoring cycle starting at 8:00 with *n* = 1, calculations begin as follows: subtract the 8:00 glucose from the 9:00 glucose; subtract the 8:05 glucose from the 9:05 glucose; subtract the 8:10 glucose from the 9:10 glucose, and so on, until subtracting the 7:00 glucose from the 8:00 glucose [48]. The fundamental principles supporting the use of the aforementioned parameters (MAD, MAG, and CONGAn) have yet to be published. To date, most studies involving these parameters have been based on follow-up examinations of diabetic patients rather than healthy subjects. Consequently, it is difficult to assign any biological relevance to them, leaving them reliant solely on mathematical calculations without considering underlying correlations. Although various blood glucose fluctuation formulas are currently available, there remains a lack of clinically practical and standardized tools for describing fluctuations. Further exploration is needed to develop glucose variability metrics applicable to populations with different metabolic characteristics.

In summary, each glycemic variability metric has its inherent strengths and limitations. SD is simple to calculate and widely used, but it is sensitive to sampling frequency and heavily influenced by mean glucose levels. CV, as a standardized measure, facilitates comparisons across populations, yet it also cannot be completely dissociated from the mean glucose value. VIM has the advantage of being independent of mean glucose levels; however, its sample-specific nature limits cross-study comparability. MAGE reflects the actual amplitude of glycemic excursions and offers good clinical intuitiveness, but its calculation is complex and relies on continuous glucose monitoring data. The J-index partially corrects the imperfect relationship between SD and mean glucose, though its clinical applicability remains insufficiently validated. Metrics such as MAG, MAD, and CONGAn, derived from continuous monitoring, can provide more detailed temporal information; nevertheless, they lack universally established clinical reference thresholds, and their biological relevance remains unclear (Table 1).

Some studies now employ the term “glucose trajectory” to quantify continuous fluctuations in glycemic control over time. This approach aids in identifying individuals whose glucose tolerance deteriorates into overt diabetes and enables predicting disease risk and adverse outcomes based on glucose trajectories. Table 2 summarizes selected studies applying trajectory models. Glucose trajectories encompass a broad range of time scales. Some studies cover multi-year changes spanning 2 to 5 years, while others focus on short-term patterns. In terms of content, some investigations examine fasting glucose variations, others analyze random blood glucose changes, and additional studies explore alterations in HbA1c and insulin resistance-related indicators. In 2019, Soshiro Ogata and colleagues used a linear mixed model to describe longitudinal fasting glucose exposure trajectories assessed at multiple time points. They explored the association between glucose trajectories and cardiovascular disease incidence (including stroke and coronary heart disease), finding a higher stroke risk among men with steeply increasing fasting glucose trajectories [49]. A study utilizing the Medical Information Marketplace (MIMIC) database from 2001 to 2019 applied latent growth mixture models to classify patients’ glucose level trajectories within 24 h of admission. It found that a moderately stable glucose level trajectory was positively associated with increased 30-day mortality in patients with acute ischemic stroke (HR, 1.28;95% CI: 1.03–1.59) [50]. A 2025 study of retired elderly individuals in China, enrolling 9426 participants aged 65 years and older, identified fasting blood glucose trajectory patterns from longitudinal data using group-based trajectory modeling (GBTM). Compared to the low–stable group, the moderate–elevated and high–stable groups demonstrated a 1.28-fold increased risk of ischemic stroke (OR = 1.28;95% CI 1.01–1.62) and 1.60-fold (HR = 1.60; 95% CI 1.20–2.15), respectively [51].

Furthermore, trajectory analysis can predict the risk of progression from normal glucose tolerance to prediabetes to overt diabetes in individuals with normal baseline glucose or glycated hemoglobin levels. Mason et al. noted in a longitudinal analysis of Pima Indians that individuals progressing to type 2 diabetes exhibited two distinct prediabetic glucose trajectories: one characterized by an initial positive linear trend followed by a steep rise in glucose levels, and another marked by an initial decline followed by a steep rise [52]. Tabák et al. conducted a prospective cohort analysis of 6538 UK civil servants without diabetes at baseline. They observed a linear increase in fasting glucose levels starting three years before diabetes diagnosis, followed by a steep secondary rise (from 5.79 mmol/L to 7.40 mmol/L). Postprandial glucose levels began to rise rapidly after a 2 h glucose tolerance test (from 7.60 mmol/L to 11.90 mmol/L), while HOMA insulin sensitivity declined sharply in the five years preceding diagnosis (to 86.7%). HOMA β-cell function increased during the 3–4 years preceding diagnosis (from 85.0% to 92.6%) and then declined prior to diagnosis (to 62.4%) [53]. Similarly, a study of Mexican diabetic populations suggested that fasting glucose levels abruptly rose to hyperglycemic ranges within a 3–5 year window preceding diabetes diagnosis [54]. In a cohort study involving 32,819 participants from Hong Kong across different diagnostic age groups, examining glucose trajectories and oral hypoglycemic responses, Ke and colleagues reported that younger-onset individuals exhibited trends of excessive glucose exposure and rapid deterioration, along with differential responses to oral hypoglycemic agents [55]. Furthermore, a study from the Whitehall II cohort examining differences in glycological traits across ethnic groups revealed that compared to Caucasians, South Asians exhibited a faster decline in insulin sensitivity and a more rapid increase in fasting glucose levels, along with more pronounced disruption of β-cell compensation, indicating significant racial differences in the natural course of diabetes [56].

Despite the accumulating evidence linking glycemic variability to the prognosis of ischemic stroke, several obstacles impede its clinical translation. First, the lack of standardization in the measurement and calculation of glycemic variability metrics—coupled with the absence of uniform clinical thresholds across studies—limits both cross-study comparability and clinical utility. Second, the current evidence base is predominantly derived from retrospective studies or post-hoc analyses, with a paucity of prospective interventional data to establish whether optimization of glycemic variability independently improves outcomes. Third, whether high glycemic variability serves as a direct pathogenic factor or merely as a prognostic marker remains unresolved. Furthermore, the existing literature disproportionately emphasizes short-term in-hospital fluctuations, leaving the relationship between post-discharge long-term glycemic trajectories and stroke prognosis inadequately explored. Lastly, the integration of glycemic variability metrics into established stroke risk assessment frameworks, as well as the development of targeted interventions, requires further investigation.

**Table 2:** Trajectory-related methods and Exposure indicator.

Study Population	Trajectory Modeling Method/Exposure Indicator	Reference
Critically ill diabetic patients undergoing coronary artery bypass grafting (CABG)	Trajectory modeling method: Latent Growth Mixture Model (LGMM). Exposure indicator: MBG (mean blood glucose), calculated from biochemical glucose records obtained every 4 h (six records at T1–T6).	[57]
Participants free of cardiovascular disease at baseline	Trajectory modeling method: Latent mixture modeling (LMM). Exposure indicator: Fasting blood glucose (measured in 2006, 2008, and 2010).	[58]
Critically ill patients without psychiatric disorders at baseline or by history	Trajectory modeling method: Latent Class Mixed Model(LCMM). Exposure indicator: 24 h glycemic trajectory after ICU admission.	[59]
Patients with sepsis	Trajectory modeling method: K-means clustering. Exposure indicator: 48 h glycemic trajectory after admission.	[60]
Patients with acute respiratory distress syndrome (ARDS)	Trajectory modeling method: Group-based trajectory modeling (GBTM). Exposure indicator: 48 h glycemic trajectory after ICU admission.	[61]
Elderly populations in China and the United Kingdom	Trajectory modeling method: Mixed-effects model. Exposure indicator: Fasting blood glucose from baseline to the year of diabetes diagnosis.	[62]
Parturient without pregestational diabetes	Trajectory modeling method: Group-based trajectory modeling (GBTM). Exposure indicator: random capillary glucose (RCG) levels throughout pregnancy.	[63]
critically ill patients who received continuous EN for ≥2 consecutive days	Trajectory modeling method: Group-based trajectory modeling (GBTM). Exposure indicator: 48 h glycemic trajectory after ICU admission.	[64]
youths with obesity	Trajectory modeling method: Latent Class Mixed Model(LCMM). Exposure indicator: plasma glucose at 10, 20, and 30 min, then every 30 min up to 180 min.	[65]
population without diabetes	Trajectory modeling method: Group-based trajectory modeling (GBTM). Exposure indicator: 24 h glycemic trajectory after admission.	[66]
critically ill heart failure (HF) patients	Trajectory modeling method: hierarchical clustering based on the dynamic time-warping algorithm. Exposure indicator: 48 h glycemic trajectory after ICU admission.	[67]

This table includes selected glucose-trajectory modelling studies from both stroke and non-stroke populations to illustrate the general methodological approaches applicable to this field.

## 5. Impact of Blood Glucose Trajectories and Oscillations on Ischemic Stroke Outcomes and Potential Mechanisms

### 5.1. Cerebral Hemorrhagic Transformation

Hemorrhagic transformation (HT) is a common spontaneous complication following endovascular recanalization therapy. In previous autopsy studies, the incidence of spontaneous HT ranged from 38% to 71%, while CT studies reported rates between 13% and 43% [68]. Its incidence depends on multiple factors including age, blood glucose levels, collateral circulation status, thrombolytic agent type, platelet count, and treatment time window [69,70]. It occurs through mechanisms including blood–brain barrier disruption, neuroinflammation induction, matrix metalloproteinase-9 (MMP-9) activation, reactive oxygen species generation, and recombinant tissue plasminogen activator (rt-PA) toxicity [71]. HT is defined and classified into four subtypes: small punctate hemorrhagic infarction (HI1), confluent punctate hemorrhagic infarction (HI2), small parenchymal hemorrhage (PH1, <30% of infarct, mild mass effect), and large parenchymal hemorrhage (PH2, >30% of infarct, marked mass effect) [72]. According to the Heidelberg criteria and the European Acute Stroke Collaborative Study classification, HT can be categorized as asymptomatic intracranial hemorrhage (ICH) or symptomatic intracranial hemorrhage (sICH). In the absence of neurological data, PH is considered equivalent to symptomatic hemorrhage, while HI is regarded as asymptomatic intracranial hemorrhage [73]. Current consensus remains divided regarding the impact of different hemorrhagic transformation subtypes on IS prognosis. As early as 1999, Fiorelli et al., based on the European Collaborative Acute Stroke Study I (ECASS I) database, demonstrated significantly increased risks of early neurological deterioration and three-month mortality following PH2. They concluded that large parenchymal hematoma (PH2) represents the sole hemorrhagic evolution likely to alter ischemic stroke prognosis [74]. A 2006 retrospective study involving 240 patients with anterior circulation ischemic stroke demonstrated that PH was a strong independent predictor of both early and late mortality, regardless of treatment modality (conservative management or intravenous thrombolysis) [75]. In 2010, based on data from the Tinzapararin Acute Ischemic Stroke Trial, indicated that asymptomatic hemorrhagic transformation (and HI type) was not associated with early or long-term functional outcomes [76]. The above studies failed to find a significant correlation between HI-type hemorrhagic transformation and IS prognosis, while other studies suggested that even small areas of minor hemorrhagic transformation could cause neurological deficits and poor recovery. A 2020 small single-center study indicated that asymptomatic intracerebral hemorrhage patients who did not receive reperfusion therapy had a higher probability of poorer mRS scores after discharge (OR, 5.99; 95% CI 1.83–19.58) [77]. Kimura et al. reported that asymptomatic intracerebral hemorrhage was an independent negative factor for early neurological improvement in 51 patients with anterior circulation ischemia undergoing intravenous thrombolysis [78]. A systematic review and meta-analysis of fourteen studies demonstrated that asymptomatic intracerebral hemorrhage was strongly associated with poor 3-month outcomes (mRS ≥ 2) exclusively in patients undergoing endovascular recanalization [79]. Hemorrhagic transformation after IS, even when considered asymptomatic in the short term, may reduce the likelihood of favorable short- and long-term outcomes following acute ischemic stroke. Therefore, actively reducing or minimizing modifiable risk factors for HT is essential to improve outcomes in ischemic stroke patients.

**Figure 1:**
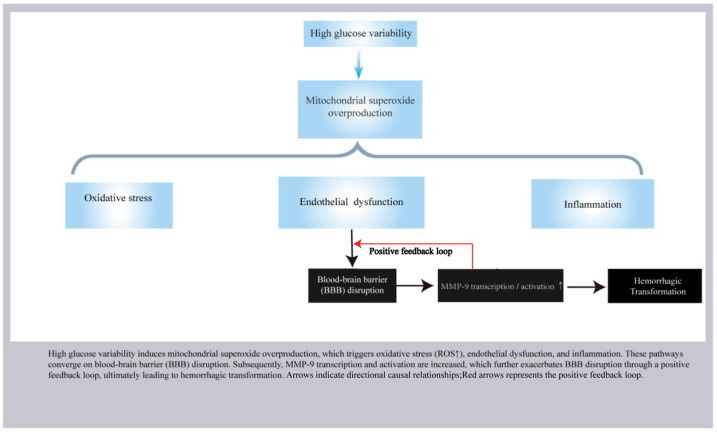
Diagram of the mechanisms linking glycemic variability to hemorrhagic transformation.

Previous studies have sparsely examined the association between glycemic variability and hemorrhagic transformation risk in AIS patients. In 2021, Kim et al. enrolled 176 patients with large-vessel occlusion AIS undergoing endovascular thrombectomy and found that short-term glycemic variability parameters, including time-rate, were significantly associated with high risk of symptomatic intracerebral hemorrhage [80], indicating that maintaining stable blood glucose is crucial for reducing hemorrhagic transformation in ischemic stroke patients. However, in 2022, Baudu and colleagues demonstrated that time-related glycemic variability within 24 h after successful endovascular recanalization was not more strongly associated with symptomatic intracerebral hemorrhage than other absolute glucose levels [81]. Compared to chronically elevated glucose levels, rapid and substantial glucose fluctuations may specifically trigger oxidative stress, endothelial cell damage, increased blood–brain barrier disruption, and heightened HT risk [82,83] (Figure 1). To date, studies examining glucose trajectories and ischemic stroke outcomes remain scarce and predominantly focus on populations with baseline type 2 diabetes. A 2025 prospective study involving 12,054 diabetic patients, with an average follow-up of 6 years, identified four distinct fasting glucose trajectories based on baseline and follow-up glucose changes: low-level stable, moderately elevated, steeply declining, and steadily rising. It highlighted the critical role of the moderately elevated and steadily rising trajectories in the occurrence of ischemic stroke among diabetic patients [84]. However, the study did not explore the association with IS prognosis.

The discrepant findings may be attributable to several factors: differences in measurement timing (pre-treatment vs. post-recanalization), recanalization status (complete recanalization may exacerbate reperfusion injury), patient characteristics (diabetes prevalence, stroke severity), small sample sizes in some studies (limited statistical power), and the use of different variability metrics (time-rate, MAGE, SD capture distinct fluctuation dimensions). The combined effect of these factors likely explains the current heterogeneity in the literature.

### 5.2. Clinical Outcomes

Patients with similar HbA1c levels may still exhibit markedly different daily glucose drift, suggesting that glycemic variability may explain the inconsistent efficacy of glucose-lowering interventions in acute stroke. In a UK trial using immediate glucose-potassium-insulin (GKI) infusion to maintain normal glucose levels post-stroke, glycemic control did not show significant beneficial effects on 90-day mortality or functional outcomes [21]. Similarly, a randomized placebo-controlled trial demonstrated that insulin therapy led to further enlargement of cerebral infarction in patients with intravascular non-reperfusion whose blood glucose levels were maintained at 7 mmol/L within 24 h after ischemic stroke [85]. These findings raise the possibility that treatment failure for hyperglycemia in ischemic stroke may be attributable to glycemic variability rather than to mean glucose levels alone.

A growing body of evidence supports that glycemic variability predicts poor outcomes in ischemic stroke patients, independent of mean glucose levels and HbA1c. One line of investigation has focused on oxidative stress pathways. Llombart et al. reported that fluorescent molecular peroxidation products, as potential molecular markers of oxidative damage, are important biomarkers for predicting endothelium-dependent neuroprotection in ischemic stroke patients [86]. Additionally, glucose variability measured by MAGE is independently associated with epigenetic regulation of the adapter protein p66Shc, a key driver of mitochondrial oxidative stress contributing to persistent vascular dysfunction in type 2 diabetes [87]. Hui et al. further confirmed that in ischemic stroke patients with type 2 diabetes, early neurological deterioration correlates with higher short-term blood glucose variability calculated using SD and MAGE [88].

Regarding mortality and functional outcomes, a systematic review and meta-analysis incorporating 10 cohort studies demonstrated that elevated acute glycemic variability significantly increased 28-day and 90-day all-cause mortality in ischemic stroke patients. This association remained consistent across different glycemic variability metrics, including CV (OR, 2.24; 95% CI 1.40–3.58), SD (OR, 2.31; 95% CI 1.70–3.13), and MAGE (OR, 3.57; 95% CI 1.44–8.85), and across different stroke subtypes [89]. In critically ill patients with acute ischemic stroke, a study based on the MIMIC database further indicated that LogCV exhibits an approximately linear correlation with severe cognitive decline and in-hospital mortality [90].

In contrast to the extensive evidence on glycemic variability, research on the association between glycemic trajectories and clinical outcomes after ischemic stroke remains limited. A 2025 multicenter retrospective study of Chinese adults aged 60 years and older used group-based trajectory modeling to identify distinct fasting glucose trajectories from 2017 to 2022. However, the relationship between glycemic trajectories and post-stroke prognosis remains largely unexplored, indicating a clear need for further investigation.

### 5.3. Post-Stroke Pain

Post-stroke pain (PSP) may occur secondary to ischemic stroke, manifesting as pain and sensory abnormalities in brain regions corresponding to the affected cerebral vasculature [91]. PSP onset coincides with stroke in 18–35% of patients, occurs within one month post-stroke in 22–42%, develops between the first month and first year in 33.9–49.0%, and manifests 12 months post-stroke in 3–8% [92]. Currently, direct evidence linking glycemic variability or trajectories to post-stroke pain is extremely scarce. To our knowledge, no published study has specifically examined the association between blood glucose fluctuations and post-stroke pain, and only a limited number of studies have investigated the relationship between absolute blood glucose levels and PSP. This represents a significant gap in the understanding of post-stroke pain etiology. However, indirect evidence from diabetic populations may provide some mechanistic insights. A recent single-center study demonstrated a strong association between chronic pain in elderly type 2 diabetic patients and parameters of glycemic variability [93]. Concurrently, prior research has shown significant correlation between glycemic fluctuations and pain severity [94]. Mechanisms linking dysregulated glucose metabolism to chronic pain may involve inflammatory stress responses, altered ion channel function, central and peripheral sensitization, and microglial activation [95]. Compared to sustained hyperglycemia, high glycemic variability readily disrupts cellular morphological stability and functional integrity, releasing pain impulse signals. A meta-analysis examining differences in elevated nitric oxide levels among hyperglycemic individuals of various ethnicities indicated that sudden blood glucose spikes promote nitric oxide production in the body [96]. NO signaling further exerts paradoxical pain sensitization and analgesic effects in neuropathic and inflammatory pain through the hypothalamic-pituitary-adrenal (HPA) axis, renin-angiotensin-aldosterone system (RAAS), calcitonin signaling, melatonin signaling, and sex hormone signaling [97]. Moreover, sudden hypoglycemia and recurrent hypoglycemic episodes can cause diffuse damage to unmyelinated and lightly myelinated nerve fibers, along with loss of superficial epidermal nerve fibers responsible for pain sensation [98]. However, no literature linking blood glucose fluctuations to post-stroke pain was retrieved, with only partial studies examining the association between absolute blood glucose levels and post-stroke pain, indicating significant unexplored territory in this area of post-stroke pain etiology.

It should be emphasized, however, that the above mechanistic discussion is largely extrapolated from diabetic neuropathic pain and general chronic pain research. Whether these pathways are directly applicable to post-stroke pain remains unknown and requires dedicated investigation. The substantial gap between indirect evidence and the absence of direct data underscores the urgent need for future studies specifically designed to evaluate the role of glycemic variability and trajectories in the development of post-stroke pain.

### 5.4. Post-Stroke Depression

Post-stroke depression (PSD) is classified under “Mood disorders due to cerebrovascular disease” in ICD-11 and is associated with increased mortality risk and adverse functional outcomes following ischemic stroke [99,100]. Previous studies report a total prevalence of PSD among stroke survivors approaching 30% [101]. However, due to variations in assessment tools, timing of evaluation, and sample characteristics—including socioeconomic and cultural diversity—the incidence of post-stroke depression lacks precise quantification. Furthermore, inconsistent definitions and the absence of accurate, unified diagnostic criteria complicate its assessment. The current mainstream definition of post-stroke depression is primarily based on DSM-5 criteria, requiring depressive mood or anhedonia lasting ≥ 2 weeks plus ≥ 4 weeks of accompanying symptoms [102]. Multiple studies have previously demonstrated an association between glucose dysregulation and post-stroke depression. A meta-analysis incorporating 21 prospective cohort studies indicated that elevated peripheral blood glucose levels during the acute stroke phase increase the risk of PSD [103]. In 2022, Liu and colleagues utilized the Metabolic Network of Depression (MENDA) database to highlight the association between altered glucose metabolism and PSD [104]. A prospective one-month study of 358 ischemic stroke patients suggested that the link between prediabetes and PSD may be attributable to abnormal glycated hemoglobin levels in prediabetic individuals [105]. However, current research on glycemic variability and trajectories in relation to post-stroke depression remains extremely limited and warrants further exploration.

### 5.5. Post-Stroke Cognitive Impairment and Dementia

Growing evidence indicates that stroke increases the risk and severity of cognitive impairment, particularly in the ischemic stroke subtype. Post-stroke cognitive impairment (PSCI) is defined as cognitive decline following a clinical cerebrovascular event. Among stroke survivors, elevated plasma levels of receptor for advanced glycation end products (RAGE)—particularly soluble RAGE and endogenous RAGE—are associated with vascular dementia [106]. Prevalence rates of PSCI vary across studies due to differences in assessment timing, study settings, population characteristics, cognitive tests employed, and cutoff thresholds. The Stroke and Cognitive Alliance (STROKOG) pooled data from 13 studies across 8 countries, revealing that 44% of hospitalized stroke patients exhibited global cognitive impairment after an average follow-up of 2 to 6 months. Between 30% and 35% of participants demonstrated deficits in specific cognitive domains, with glucose dysregulation identified as an independent risk factor [107]. According to several longitudinal studies, approximately 20% of patients with a first stroke or prior history develop early-onset post-stroke dementia, while 4.4% to 23.9% develop delayed-onset post-stroke dementia [108,109]. Although stroke survivors’ cognitive function significantly improved over 3 to 12 months, a 35.4% prevalence remained at 12 months [110]. Furthermore, the risk of dementia was highest one year post-stroke, with the increased risk persisting even after 10 years [111]. A cohort study involving 354 IS patients with 3-month follow-up indicated that glycemic variability was a significant predictor of PSCI in the diabetic subgroup [112]. Studies using the difference between initial admission blood glucose and mean blood glucose (eAG) to represent glycemic variability found that compared to the non-elevated group, greater variability was associated with higher PSCI risk and significantly poorer performance in frontal lobe and memory domains [113]. In 2024, Zhou et al. based on a prospective cohort study of 209 IS patients, indicated that each 1-point increase in the glycemic MAGE score was associated with a 76.4% higher probability of cognitive decline [114]. Research on the relationship between glycemic trajectories and PSCI remains limited and warrants further exploration.

## 6. Effective Management Strategies for Glucose Trajectories and Oscillations

Given the adverse impact of blood glucose trajectories and oscillations on AIS prognosis, more personalized glucose management should be adopted for IS patients. In terms of monitoring strategies, continuous glucose monitoring (CGM) is increasingly utilized in clinical practice, and prospective clinical studies are increasingly employing CGM devices to collect data and assess participants’ glucose profiles. Although the efficacy of CGM remains unclear for insulin-dependent versus non-insulin-dependent individuals, guidelines recommend CGM use for insulin-treated type 2 diabetes patients to aid in real-time identification of hypoglycemic episodes and glucose drift [115]. A systematic review and meta-analysis based on 1384 CGM and 4902 intermittent glucose monitoring cases demonstrated that CGM significantly improves HbA1c in adults with type 2 diabetes [116], enabling personalized management for patients with poor long-term glycemic control.

Regarding lifestyle interventions, some studies evaluated the association between CGM use and dietary changes via food diaries or web-based 24 h dietary recall, finding that CGM use significantly reduced daily total calorie intake, enhanced dietary control, and increased awareness of dietary intake, body composition, and specific metabolic indicators [117,118]. Taylor et al. enrolled 20 obese patients with type 2 diabetes, establishing a CGM wear group and an “offline blind” control group. Their findings suggest CGM can significantly reduce blood glucose variability by six-fold through facilitating a low-carbohydrate lifestyle transition [119]. Time in Range (TIR), Time Below Range (TBR), and Time Above Range (TAR) serve as quantitative metrics describing glycemic trajectory quality. A recent meta-analysis indicated that using CGM as a behavioral intervention tool increased TIR by 7.4% compared to controls, exceeding the 5% clinical significance threshold [120]. This aligns with the findings of a meta-analysis by Molfetta et al. based on 22 studies, which reported a 5.6% increase in TIR from baseline following CGM use in patients with T1DM or T2DM [121]. A large cohort study involving 22,006 patients further demonstrated that CGM users significantly increased TIR by reducing glycemic variability [122]. These studies suggest beneficial effects of CGM application on achieving favorable glycemic trajectories.

Beyond monitoring and lifestyle modifications, pharmacological agents also play a role in reducing glucose oscillations. Glucagon-like peptide-1 analogues and dipeptidyl peptidase-4 inhibitors have been shown to significantly influence blood glucose variability in type 2 diabetes mellitus patients [36]. Sodium-glucose cotransporter 2 inhibitors, beyond controlling blood glucose, significantly reduce glycemic variability in untreated type 2 diabetic patients [40]. The development of rapid-acting and long-acting insulins has also positively impacted glycemic oscillation control [37,38].

However, the aforementioned evidence primarily originates from stable outpatient diabetes management settings. Integrating CGM into glucose management for AIS patients presents unique stroke-specific challenges and evidence gaps: First, acute stress states, dysphagia, and variable nutritional support regimens lead to more severe and unpredictable glucose fluctuations. Second, the optimal target range for acute stroke glucose control (thresholds for TIR, TAR, TBR) remains unclear. Moreover, whether real-time CGM feedback can promptly guide clinical decisions (e.g., insulin titration) to shape beneficial trajectories like the “high-level rapid decline” or “moderate variability” observed in AIS patients remains to be confirmed. Finally, CGM has practical barriers in terms of cost, accuracy, and user-perceived operational inconvenience.

## 7. Summary and Outlook

In summary, dynamic glucose management—encompassing trajectory and variability—has emerged as a critical determinant of stroke prognosis. Substantial evidence indicates that abnormal glucose trajectories (e.g., persistent elevation) and high variability not only exacerbate brain injury through increased oxidative stress, endothelial dysfunction, and neuroinflammation but are also independently associated with adverse outcomes including hemorrhagic transformation, early neurological deterioration, post-stroke pain, depression, and cognitive impairment. However, most available trajectory evidence currently originates from populations with diabetes, particularly type 2 diabetes, which may limit its generalizability to non-diabetic ischemic stroke patients. This strongly suggests that acute stroke glucose management should transcend controlling isolated blood glucose values and instead pursue optimizing dynamic glucose patterns. However, significant research gaps remain. First, most evidence focuses on glucose variability, while studies examining how short-term in-hospital glucose trajectories influence acute IS outcomes are scarce, with limited exploration of underlying mechanisms. Second, while numerous metrics exist for assessing glucose dynamics (e.g., VIM, MAGE, TIR), the lack of standardized definitions and clinical thresholds for these indicators remains a major obstacle to cross-study comparison and clinical translation, as their interpretation varies considerably across studies. More critically, while CGM technology demonstrates immense potential in diabetes management for shaping optimal glucose trajectories and reducing variability, its translation into acute stroke care faces multiple challenges. These include target setting under acute stress conditions, integration into clinical decision-making, and validation of cost-effectiveness. Future research should leverage CGM and similar technologies to intensively collect acute stroke glucose data, employ trajectory models (e.g., GBTM) to identify glucose dynamic subtypes clearly associated with clinical hard endpoints (e.g., END, 90-day mRS), elucidate underlying pathophysiological mechanisms (e.g., blood–brain barrier integrity, microglial activation), and thereby explore establishing glucose dynamic control targets tailored for the acute stroke phase with the goal of improving prognosis. Specifically, future research should prioritize: (1) standardization of glycemic variability metrics—systematic comparison of SD, CV, MAGE, and VIM in large stroke cohorts to determine the optimal indicator and clinical thresholds; (2) optimization of CGM in acute stroke—defining appropriate timing, duration, and TIR/TAR/TBR targets and establishing how real-time data can guide insulin titration; (3) integration of trajectory modeling—combining CGM data with GBTM/LGMM to develop real-time warning systems and explore subtype-specific treatment responses; and (4) prospective and interventional studies—conducting well-designed cohort studies and RCTs to evaluate whether interventions targeting high variability or adverse trajectories independently improve long-term outcomes.

It should be acknowledged that, as this was a narrative review, we did not perform a formal quality assessment or risk-of-bias analysis of the included studies. While this approach is consistent with the narrative nature of this review, it represents a limitation that should be considered when interpreting the findings.

## 8. Clinical Implications and Remaining Gaps

Despite growing evidence linking glycemic variability to stroke prognosis, its translation into clinical practice remains limited. Currently, routine assessment of glycemic variability in all ischemic stroke patients is not supported by high-level evidence, given the predominance of retrospective studies and the lack of prospective interventional data. However, monitoring parameters such as CV or MAGE may provide adjunctive prognostic information in diabetic patients or those receiving intensive insulin therapy.

Similarly, CGM cannot yet be universally recommended for all stroke patients, but may be reasonably considered in selected subgroups, including those with labile glucose control, those on intensive insulin therapy, and critically ill patients with frequent fluctuations.

Several unresolved gaps prevent broader clinical integration: (1) lack of standardized metrics and cutoff values; (2) unclear causality (direct pathogenic factor vs. prognostic marker); (3) absence of prospective trials targeting variability reduction; (4) insufficient evidence on post-discharge long-term trajectories; and (5) uncertain integration into existing risk stratification tools. Addressing these issues remains a priority before glycemic variability-based management can be incorporated into routine stroke care.

## 9. Conclusions

This review highlights that glycemic trajectories and variability are independently associated with adverse ischemic stroke outcomes, including hemorrhagic transformation, neurological deterioration, post-stroke pain, depression, and cognitive impairment. Future research should prioritize well-designed prospective studies and integrate continuous glucose monitoring with trajectory modeling to establish personalized glucose management strategies, ultimately translating these findings into improved clinical practice for stroke patients.

## Figures and Tables

**Table 1:** Comparison of glycemic variability metrics.

Metric	Clinical Interpretation	Advantages/Limitations	Evidence for Stroke
SD	Dispersion of glucose values around the mean	Advantages: Simple to calculate; widely used; included in most variability studies. Limitations: Sensitive to sampling frequency; heavily influenced by mean glucose level.	Commonly used; significant predictor of 3-month mortality in severe stroke [44].
CV	Standardized measure of variability independent of units	Advantages: Enables direct comparisons across populations with different mean glucose levels. Limitations: Still partially correlated with mean glucose; cannot be completely dissociated from mean value.	Significant predictor of 3-month mortality in severe stroke [44]; associated with total stroke and ischemic stroke risk [45].
VIM	Variability measure independent of mean level	Advantages: Not correlated with mean glucose level. Limitations: Sample-specific; limits cross-study comparability.	Dose–response relationship with stroke risk in diabetic patients [43].
MAGE	Actual amplitude of glycemic excursions	Advantages: Clinically intuitive; reflects real glucose fluctuation amplitude. Limitations: Complex calculation; requires CGM data; time-consuming.	Significant predictor of 3-month mortality in severe stroke [44].
J-index	SD incorporated with mean glucose	Advantages: Partially corrects the imperfect relationship between SD and mean. Limitations: Clinical applicability remains insufficiently validated.	Limited evidence in stroke populations.
MAG	Sum of differences in glucose curve per 24 h divided by hours between first and last measurements	Advantages: Provides detailed temporal information. Limitations: Lacks established clinical reference thresholds; biological relevance unclear.	Negatively correlated with in-hospital neurological improvement [42].
CONGAn	SD of differences between current glucose and glucose *n* hours prior	Advantages: Provides time-lag specific variability assessment. Limitations: Requires complete tracking period; choice of *n* is arbitrary; lacks biological rationale.	Limited evidence in stroke populations.

## Data Availability

Data sharing is not applicable to this article as no datasets were generated or analyzed during the current study.
